# Using the VIA Classification to Advance a Psychological Science of Virtue

**DOI:** 10.3389/fpsyg.2020.565953

**Published:** 2020-12-07

**Authors:** Robert E. McGrath, Mitch Brown

**Affiliations:** ^1^School of Psychology and Counseling, Fairleigh Dickinson University, Teaneck, NJ, United States; ^2^Department of Psychological Science, University of Arkansas, Fayetteville, AR, United States

**Keywords:** virtue, character strengths, flourishing, evolutionary psychology, practical wisdom

## Abstract

The VIA Classification of Character Strengths and Virtue has received substantial attention since its inception as a model of 24 dimensions of positive human functioning, but less so as a potential contributor to a psychological science on the nature of virtue. The current paper presents an overview of how this classification could serve to advance the science of virtue. Specifically, we summarize previous research on the dimensional versus categorical characterization of virtue, and on the identification of cardinal virtues. We give particular attention to the three-dimensional model of cardinal virtues that includes moral, self-regulatory, and intellectual domains. We also discuss the possibility that these three clusters be treated as fundamental elements of a virtue model, meaning that they clearly and directly contribute to both individual and communal flourishing across various cultures. This discussion includes a summary of previous speculations about the evolution of adaptations underlying the human capacity for using behavioral repertoires associated with the three virtues, as well as discussing ways in which they simultaneously enhance community and individual, in the last case focusing particularly on evidence concerning mating potential. We then discuss the relationship between the evolutionary perspective on virtues and Aristotle’s concept of the reciprocity of the virtues. Finally, we provide speculations about the nature of practical wisdom. While accepting the potential value of future revisions to the VIA model, that model even under its current conditions has the potential to generate a number of intriguing and testable hypotheses about the nature of virtue.

## Introduction

*It is not profitable for us at present to do moral philosophy; that should be laid aside at any rate until we have an adequate philosophy of psychology, in which we are conspicuously lacking.* – (Anscombe (1958), p. 1)

The VIA Classification of Character Strengths and Virtues (Peterson and Seligman, 2004) was intended as the starting point for a science of positive human functioning. The model consists of 24 character strengths that were conceptualized as reflections of six virtues. One aspect of the model that has not received as much attention as it deserves is the potential for using the VIA Classification as a tool for the scientific study of virtue.

Virtues can be conceptualized as personal traits that are in general practice both personally and communally valuable, such as the tendency to be kind or critically evaluate information. A science of virtue would therefore focus on issues such as the measurement of these traits, how they develop, and how their development can be encouraged (for further details, see Fowers et al., in press).

Is a science of virtue a worthwhile endeavor? A critical analysis of the concept of virtue could reasonably argue that virtue must prove itself to be more than a bromide of the Greco–Roman world that Christians found useful, and as a result has infiltrated modern Western moral philosophy simply because of heritage. The reality is that virtue ethics offers a distinct approach to thinking about the moral and collective role of the individual, one that is likely to prove particularly amenable to a scientific, and psychological, analysis.

It is noteworthy that we are not the only psychologists calling for the scientific study of virtues as person traits (Cokelet and Fowers, 2019; Fowers et al., in press). Several points can be raised to support virtue as a worthwhile topic of scientific and psychological study. First, virtue ethics is primarily an inquiry into the nature of the moral actor rather than the moral act. Where deontological and utilitarian perspectives were founded for the purpose of identifying moral rules, modern virtue ethics more than anything else is about how the actor decides what it means to act well. Similarly, where the deontological and utilitarian perspectives focus specifically on understanding morality, in developing their conception of virtue the Greeks and Romans were more interested in an ethic for a good life, a life of self and communal enhancement. This perspective includes an interest in attributes that are not strictly moral but that still advance both the individual and the individual’s community, the classic example being Aristotle’s interest in intellectual as well as moral virtues. A comprehensive virtue ethics will require considering how a person makes virtuous decisions in complex, ambiguous, and uncertain real-world circumstances that involve competing considerations. Clearly this falls within the purview of a scientific psychology interested in contributing to both the social and the individual good.

In this article, we suggest several ways in which the VIA Classification can offer and already has been used to evaluate some valuable hypotheses relevant to the development of a psychology of virtue, i.e., an empirically founded theory of what represents a relatively virtuous (personally enriching, socially admirable, and communally desirable) orientation to life. The following topics will be addressed primarily from the perspective of the VIA Classification:

1. The dimensional versus categorical conceptualization of virtue.
2. Toward a hierarchical taxonomy of virtues.
3. The evolutionary perspective on fundamental virtues.
4. Reciprocity of the virtues.
5. The nature of practical wisdom.

## Preliminary Issues

Before turning to specific topics, though, three issues should be addressed. (1) The VIA Classification assumes a hierarchical relationship between constructs identified as character strengths and constructs identified as virtues. Where the VIA Classification distinguishes between broader virtues and more specific character strengths, both levels are relevant in the context of virtue ethics. In his discussion of virtue ethics, the philosopher Russell (2012) has offered an alternative lexicon of *cardinal virtues* (corresponding to the VIA virtues) and *subordinate virtues* (the character strengths). Still a third set of terms can be found in personality psychology, where hierarchical structuring is described in terms of *domains* and *facets* (Costa and McCrae, 1995). The choice of terminology is somewhat arbitrary and will vary in this article depending on which framework is most useful at that point.

(2) Our critical analyst could fairly ask whether the VIA character strengths provide a sound foundation for empirical explorations on the nature of virtue. Though there is consensus among virtue theorists that virtue ethics can be grounded in a set of personal attributes called the virtues, no authoritative description of this set has emerged in the literature. For example, Table 1 is a sampling of virtue lists just since the beginning of the 20th century, and many others are available. There have even been discussions among philosophers of whether a listing of “the” virtues is possible or necessary.

**TABLE 1:** Taxonomies of virtue since the 20th century.

**Bennett (1995)**	**Cawley et al. (2000)**	**Comte-Sponville (2001)**	**Dahlsgaard et al. (2005)**	**Erikson (1964)**	**Moore (1903)**	**Rand (1984)**
Compassion	Empathy	Compassion	Courage	Care	Aesthetic Enjoyment	Honesty
Courage	Order	Courage	Humanity	Competence	Interpersonal Enjoyment	Independence
Faith	Resourcefulness	Fidelity	Justice	Fidelity		Integrity
Friendship	Serenity	Generosity	Temperance	Hope		Justice
Honesty		Gentleness	Transcendence	Love		Pride
Loyalty		Good Faith	Wisdom and Knowledge	Purpose		Productivity
Perseverance		Gratitude		Will		Rationality
Responsibility		Humility		Wisdom		
Self-Discipline		Humor				
Work		Justice				
		Love				
		Mercy				
		Politeness				
		Prudence				
		Purity				
		Simplicity				
		Temperance				
		Tolerance				

Here we see an important epistemological difference between philosophical and psychological approaches to virtue. From the former perspective, it is still possible to draw analytic conclusions about the nature of the virtues without an established enumeration of the virtues, whereas a scientific psychology of virtue requires a bedrock of well-defined constructs. The lack of an established virtue list potentially interferes with the development of a science of virtue in several ways:

1. If it is agreed that virtue ethics is founded in a set of person attributes deserving of being called virtues, the enumeration of those attributes will play an important role in the testing of empirical hypotheses about virtue. Parallels can be drawn to scientific advances made possible by the periodic table of the elements, the Linnaean approach to biological classification, or (closer to home) the five-factor model of personality.
2. If being virtuous means acting according to the virtues, but the list of virtues is indefinite, clear hypotheses about what it means to act virtuously can be impossible (see Russell, 2012, for a discussion of this issue).
3. If different researchers rely on different conceptualizations of the key dimensions of virtue, the potential for a cumulative science of virtue is reduced. Research that tests a hypothesis about virtue using one model of the virtues may have little to say about the validity of that hypothesis for other virtue models, or for virtue theory in general. For example, various educational programs have been created that focus on virtue development in students, but it is problematic to use evidence for one program as evidence for the field in general if the target constructs differ markedly.

These concerns can be overstated. Review of the virtue lists in Table 1 demonstrates a substantial degree of overlap, suggesting some informal consensus on cardinal traits. That said, the examples provided in our first bullet point above demonstrates the degree to which a reasonable taxonomy has proven a valuable empirical tool in other contexts.

Even if one accepts the importance of a shared virtue list for achieving the accumulation of knowledge in a science of virtue, the question remains whether the VIA character strengths represent an adequate starting point for developing such a list. For example, its comprehensiveness is difficult to establish, especially as some enumerations of virtues have been substantially longer (e.g., Hume, 1751/2010)^1^.

In response, it can be noted that few attempts at the development of a virtue list have involved so many sources of input or been so transparently and collaboratively developed as the 24 VIA strengths. More than 50 experts in positive human functioning contributed to the project, multiple literature reviews were conducted to support the process, and 13 of the leading experts in this field were involved in decision-making (Peterson and Seligman, 2004). Explicit criteria were generated for identifying which character strength candidates were retained in the final list. In contrast, most other lists have been proposed without any justification or vetting. A recent study in which homeless youth were invited to list personal characteristics that were particularly meaningful to them in their attempts to thrive or cope with life’s challenges found that 98% of responses could be categorized according to the 24 VIA character strengths (Cooley et al., 2019), providing some empirical evidence for their comprehensiveness. On the other hand, a recent study examining how ordinary people characterize virtue revealed 10 of 24 VIA strengths were never mentioned (Gulliford et al., in press). The omissions seemed to represent a combination of instances in which the emphasis on positive functioning in the identification of the VIA strengths resulted in the inclusion of constructs not typically associated with virtue (e.g., teamwork was absent), variations in how experts and ordinary people are likely to conceptualize virtue (e.g., justice was absent), and terms that partially overlap (e.g., social intelligence and empathy/sympathy).

Assuming more work can be done to develop a sufficiently comprehensive set of virtues, it is worth noting that a taxonomy need not be perfected before it can be used to make important contributions. Methods of classifying life on earth have matured over time, and that classification system remains incomplete even today. If the VIA character strengths can be considered a reasonable starting point for a catalog of important virtues, then they can serve the purpose of testing hypotheses about the nature of virtue even while recognizing that future revisions of the model are possible that could require modifying the conclusions drawn.

(3) Aristotle was one of the first great systematic observers of nature in history. As a result, he generated several important hypotheses about practical ethics, as he also did about biology. Biologists took some of those hypotheses as a basis for empirical inquiries, retaining or rejecting his proposals as called for by the evidence. Some modern writers on virtue seem to have adopted a different orientation to his work, assuming elements of Aristotelian virtue theory are essential based solely on his authority, or rejecting propositions because they are inconsistent with Aristotelian thought. In a science of virtue, Aristotelian propositions must be required to stand or fall on their own merits. In what follows we will refer to Aristotelian concepts, but we intend those references to serve solely as background to our inquiries into the nature of virtue.

## Variation in Virtue: Categorical or Dimensional?

For example, in his *Nicomachean Ethics* Aristotle discussed his concept of the *phronimos*, the individual who is a skilled judge of questions about the good, someone to whom others are likely to turn for guidance on such issues. In doing so he reinforced a Greek–and later Roman–tradition of seeing the virtuous as a distinct class of individuals. Aristotle expanded on this vision of the distinctly virtuous person when he distinguished between the continent person (virtuous despite temptations to act invirtuously) and the virtuous person (whose desires and behaviors are consistently virtuous). This question of whether there are people who are categorically superior in their virtuous judgments is a good example of where quantitative psychology can offer an empirically informed if not authoritative conclusion.

A variety of statistical methods have been developed to evaluate whether interpersonal variation should be understood as primarily categorical or quantitative. Two studies have now been completed using scores on the VIA Inventory of Strengths (VIA-IS; Peterson and Seligman, 2004) to evaluate whether there are meaningful categorical distinctions in the VIA character strengths (McGrath et al., 2010; Berger and McGrath, 2018). Using very different analytic strategies, both drew the same conclusion: there is no evidence that (at least based on individuals who completed the VIA-IS) there exists a distinctly virtuous class of individuals.

As with any first-generation set of findings, they must be interpreted with caution. It is possible the class of individuals meriting the label of *phronimos* is vanishingly small, though that raises questions about the practical value of discussing them. It is also possible the samples for these studies, drawn from two websites that offer completion of and feedback on the VIA-IS for free, included an unusually small subset of the *phronimoi*, though one must then question where is one to find them in sufficient concentrations that they are detectable. With these caveats in mind, the burden would seem to fall upon those who believe in the qualitatively virtuous to demonstrate their existence^2^.

Assuming this is a valid conclusion, what are its practical implications? Most immediately, in the coming sections we will generally refer to individuals high in virtue or relatively virtuous, rather than to virtuous individuals. More broadly, rejecting the archetype of the virtuous person except as an ideal complicates the identification of moral exemplars, because it suggests no one is immune to temptation. On the other hand, it raises the question of whether Aristotle’s description of virtue immune to temptation is a fictionalized ideal, or at best only possible in rarefied settings such as monastic orders. On a more practical level, it could be used to argue that even individuals identified as relatively virtuous should not become complacent about their virtue but should recognize that maintaining a virtuous life requires continuing commitment and self-reflection. There is something challenging in the suggestion that virtue is not a status one achieves, but a status one can only hope to achieve (also see Cokelet and Fowers, 2019).

## A Taxonomy of Virtue

As noted previously, Aristotle suggested the virtues could be organized into two groups, the moral and the intellectual. He was not the first to consider ordinality in the virtues. Plato earlier suggested four cardinal virtues that encompassed a “swarm” of more specific virtues: wisdom, temperance, courage, and justice. In the same way that virtue lists merit objective justification, though, hierarchies of virtues developed for psychological purposes should be based on empirical evidence.

To date, four teams of psychologists have attempted the empirical development of a set of cardinal virtues. Two were based on lexical methods that proved important to the development of the five-factor model of personality. Cawley et al. (2000) identified 140 self-descriptive English language terms drawn from the dictionary that reflected what a person “ought” to be or do. Factor analysis of student self-ratings on these terms suggested four latent dimensions, labeled empathy, order, resourcefulness, and serenity. De Raad and van Oudenhoven (2011) collected 153 Dutch terms for moral traits. Factor analytic methods were again applied to quantitative ratings on the traits, mainly of college students. They identified two primary clusters of virtues, called sociability and ambition.

The third attempt was part of the development of the VIA Classification (Dahlsgaard et al., 2005). This was a review of traditional moral texts from seven different cultures looking for common themes. Though still empirical, it was the only effort that was not quantitative, raising concerns about objectivity in the identification of cardinal traits. These authors generated the list of six virtues that was incorporated into the VIA Classification: wisdom and knowledge, courage, humanity, justice, temperance, and transcendence. In introducing the Classification, Peterson and Seligman (2004) explicitly opined that quantitative research might not support this model.

Factor analytic studies with the VIA-IS in fact did not converge with these six factors. However, subsequent studies have found that when the solution is restricted to three factors, the solutions are equivalent across different measures of the VIA character strengths, populations, and analytic methods (McGrath, 2015; McGrath et al., 2018; McGrath, in press). These three factors have been labeled caring, inquisitiveness, and self-control, terms that were chosen because they were unassigned in the context of the VIA Classification. As cardinal variables, they encompass the moral, intellectual, and self-regulatory domains of character strengths (McGrath, Unpublished). Some cross-cultural evidence exists for these three domains, suggesting a degree of universality for these domains and bolstering an argument of these virtues having an evolutionary basis to them. Independent factor analytic studies involving residents of the United States, Switzerland, China, and Brazil all produce the same structure (McGrath et al., 2018), as did studies using other measures of the 24 strengths besides the VIA-IS. To the extent that the VIA Classification character strengths can be considered a relatively comprehensive representation of positive personal traits, these three virtues seem to offer the most defensible model of how character traits tend to cluster. That said, the 24 strengths were not chosen based on their coherence, so some strengths such as humor or humility are not well-represented by this structure.

What is striking here is the degree of overlap across four attempts to define a set of cardinal virtues inductively using very different approaches. Cawley et al.’s (2000) empathy, order, and resourcefulness correspond quite well with the caring, self-control, and inquisitiveness factors, respectively. Their inclusion of a serenity factor likely reflects their decision to focus on what one “ought” to do without explicitly limiting it to traits with both direct personal and communal value, which is a traditional expectation of virtues. Similarly, De Raad and van Oudenhoven’s (2011) sociability and ambition clusters are consistent with the caring and self-control factors; their failure to identify an inquisitive cluster may well reflect their restriction to “moral” traits (in fact, Aristotle’s moral virtues included traits reflecting strictly moral as well as self-regulatory virtues). The three-virtue model differs from that of the original VIA Classification in terms of the combination of courage and temperance in the self-control virtues, and humanity and justice in the caring cluster, and the omission of transcendence as a virtue cluster. McGrath (Unpublished) discussed the implications of this last variation.

The differences in the two systems associated with the VIA Classification raise important points to understand about the nature of taxonomies. Taxonomies can serve both ontological and heuristic purposes. In terms of the latter, different levels of granularity may be appropriate to different contexts. The modern Linnaean classification system allows for at least eight different levels of generality. In the context of virtue, it may well be the case that at times the distinction between courage and temperance will be important, at others the self-control domain as a whole will be of interest. De Raad and van Oudenhoven (2011) suggested further differentiation of each of their two clusters into three subsets of virtues. Similarly, there may be times that the goal is to capture the whole spectrum of traits recommended for personal development, in which case the inclusion of serenity can be included important; similar conclusions could be drawn about transcendence. As a practical point, the six-virtue VIA model may be more useful in the context of organizing feedback from test results, since each character strength is associated with one and only one virtue; the empirical relationships between the strengths and the three virtues are messier. The next section discusses a context in which the latter structure is more useful. The point is that a taxonomic system can be used flexibly, with different purposes suggesting different choices among the available options.

## Evolutionary Adaptiveness at the Individual and Communal Levels

McGrath (in press) suggested the three cardinal virtues described in the previous section are also *fundamental*: virtue domains that are so clearly and directly related to the flourishing of individuals and communities that there is an evolutionary basis for their emergence. Historically, individuals faced various problems related to survival and reproduction. Those possessing traits that would pose a survival advantage to their group, and traits that would increase the likelihood of personally reproducing, were at an increased likelihood of the survival of their genes. Although this process is typically described in relation to physical traits such as erect posture to help navigate savannas effectively (Dean, 2000), it has been argued that psychological processes such as biases and emotions similarly emerged to solve survival and reproductive problems (Cosmides and Tooby, 1992). These adaptations ostensibly include socially desirable personality traits, including virtuous tendencies, that would have been preferred by group members (Buss, 2009; Lukaszewski, 2013; but see Tooby and Cosmides, 1990).

The evolutionary understanding of psychological processes has several implications for cross-cultural recognition of the three domains. It suggests that attitudes and behaviors consistent with the three domains should emerge across a wide variety of environments and cultures, that a wide variety of cultural groups will value attitudes and behaviors consistent with the three domains, and that terms consistent with the three domains should emerge in many folk languages. Similarly, various cultures’ virtue concepts (markers of the desirable group member) should reflect themes associated with these domains^3^. In support of the hypothesis that the three domains have deep adaptive value, McGrath (in press) identified abilities across a variety of species, some of which had evolved multiple times, that allow for achieving goals associated with the three domains. In the following sections, we will summarize the adaptations discussed by McGrath. We will then expand on McGrath’s previous discussion of this topic, by reviewing various ways in which the three virtues contribute both to communal flourishing and to individual flourishing, with particular emphasis on various speculations about the ways in which they can contribute to reproductive success.

### Evolutionary Value of the Moral Domain

There is a considerable research discussing the ancestral origins of behavioral and phenomenological contributors to the moral domain. Humans are an intensely social species whose survival has been contingent upon group living and cooperation among group members (Baumeister and Leary, 1995; Boyd and Richerson, 2005). Selection likely favored groups capable of engaging in social exchanges that rewarded altruistic behaviors and punished selfishness (Cosmides and Tooby, 2006). The adaptive response to these selection pressures emerged as reciprocal altruism between genetically unrelated conspecifics (Trivers, 1971), kin selection among those who were related (Hamilton, 1964), and prosocial behaviors that enhanced the inclusive fitness of an individual’s own genes (Dawkins, 1976). Rules of morality may have thus evolved to facilitate the prosociality necessary for group living, wherein a social group codified the appropriate treatment of others based on how to optimize reciprocal altruism and punish free riders (Krebs, 2008; Fowers, 2015).

Because of how critical the moral domain is in supporting group living, presenting one’s self as prosocial and capable of engaging with others potentially contributes to personal acceptance, esteem, and access to resources and mates. Recent findings have indicated that morality itself can serve as an interpersonal signal that provides information to others of one’s ability to adhere to socially prescribed conventions that contribute to survival and reproductive goals. Individuals espousing a largely deontological moral ethic rooted in an aversion to directly harming others, even if that harm leads to a greater good (i.e., utilitarianism), are selected more frequently as interaction partners, with observers subsequently cooperating more with them in trust games (Everett et al., 2016; Bostyn and Roets, 2017b; Sacco et al., 2017).

This preference for individuals who exhibit cooperative behaviors appears to be rooted in a tendency to perceive such individuals as especially unlikely to allow harm to befall others (Rom et al., 2017). Conversely, individuals who appear particularly calculating in their decisions to cooperate with others are distrusted and not selected for further interactions (Jordan et al., 2016; Sacco et al., 2017). Humans seem particularly aware of the impact these factors have on how they are perceived by others, as individuals increase their endorsement of conventional morality in the presence of others, particularly those espousing conventional morality themselves (Bostyn and Roets, 2017a; Jordan and Rand, 2020).

In choosing long-term mates versus a mate for a single sexual encounter, individuals prioritize kindness (Buss and Schmitt, 1993; Li et al., 2013). Some have suggested this kindness preference provides an historical adaptive advantage for both men and women, albeit more so for women (Trivers, 1972; Symons, 1979). Women’s kindness might implicate them as more willing to provide necessary infant care, whereas men’s kindness could indicate they are more willing to provide resources for their mates and offspring. Selection of caring mates may also have facilitated biparental investment, thus offsetting the extensive care required for young human children by increasing the likelihood they would survive into adulthood and reproduce (Puts, 2016). Previous findings have demonstrated that individuals whose behavioral repertoires connote various components of care (e.g., altruism, aversion to harm) are more desirable long-term mates and appear especially disinterested in infidelity (Barclay, 2010; Farrelly, 2013; Brown and Sacco, 2019). Such displays of benevolence are most prevalent when the motivation to acquire a long-term mate is heightened. This may be particularly true for male signaling because of women’s greater attention to cues suggesting moral character (Bleske-Rechek et al., 2006; Griskevicius et al., 2007). Recent work from our research program further indicates that men and women prefer a long-term mate whose behavioral repertoire connotes valuing of the caring domain of virtue (Brown et al., 2020).

### Evolutionary Value of the Self-Regulatory Domain

Whereas the moral domain focuses on investment in others outside the self, the self-regulatory domain has to do with the organization of behavior in the service of goal achievement. McGrath (in press) saw precursors to human self-regulatory behaviors in various capacities across species for behavioral inhibition and behavioral integration. The former refers to the suppression of automatic or prepotent behaviors, whereas integration refers to the capacity to plan and implement complex behaviors to facilitate achievement of a longer-term goal. It has been posited that greater self-regulatory abilities are associated with the slower metabolism and longer lifespans of larger organisms (Stevens, 2014). When primed with ecological harshness, individuals from economically advantaged backgrounds are especially willing to forego immediate gratification in the service of attaining larger future rewards, which has been argued to ensure one has continued access to resources for future reproductive opportunities (Griskevicius et al., 2011a,b; Hill et al., 2013). This delayed gratification is less apparent among those living in chronically harsh environments, which are also associated with earlier reproductive ages and higher reproductive rates (e.g., Brumbach et al., 2009). Taken together, these findings suggest a possible origin of the self-regulatory domain that is contingent upon ecological factors determining whether self-control is important to individual flourishing.

The coordinated efforts resulting from self-regulation may have further afforded individuals the opportunity to navigate the complex interactions of group living, which could serve to increase access to resources. This access to resources could have been particularly attractive to females where males compete for access to mates (including humans, cross-culturally) who are seeking a long-term partner with considerable access to resources (Kenrick et al., 1993; Zhang et al., 2019; Walter et al., 2020). Those who demonstrate greater self-regulation may also have been perceived as less prone to infidelity (Gailliot and Baumeister, 2007), which reduces concerns about reproductive issues such as paternal uncertainty (Buss and Schmitt, 1993; Platek and Shackelford, 2006). For example, the personality construct of conscientiousness, which correlates well with the self-regulatory virtue domain (McGrath et al., 2018), has been associated with a proclivity toward monogamous mating (Schmitt and Shackelford, 2008). Prospective mates exhibiting considerable self-control were preferred in a long-term mating context, with individuals reporting a dispositional interest in monogamy having a particular strong interest in these mates (Brown et al., 2020).

### Evolutionary Value of the Intellectual Domain

The adaptive function of inquisitiveness is to reduce uncertainty within the environment. In fact, environmental exploration is the most ancient adaptation, and most basic contributor to species flourishing, of any adaptation underlying the three virtue domains (McGrath, in press). In more complex species, inquisitiveness is closely associated with investigating one’s environment without specific purposes, which is associated phenomenologically with curiosity. Exploration for mammals and other large-brained organisms is intrinsically rewarding and seems to increase inclusive fitness despite its non-directive quality because of the greater likelihood of identifying fitness-enhancing opportunities such as food, resources, and mates (Réale et al., 2007; Singh et al., 2010). In humans, this process can ultimately result in the formalization of information as propositions or statements of belief.

Non-directive searching provides information that can prove useful if the environmental circumstances change. Such exploration makes it possible to modify behavior in response to additional information. In the case of humans, incorporating information even though it has no immediate value enhances the potential for successful responding in future novel situations. The emergence of science as the most effective method of accurate information gathering in humans has been particularly contributory to our mastery of the full spectrum of environments available on our planet, as well as explorations of extraterrestrial environments with the possibility of future mastery.

Although not necessarily observed or valued in all cultures to the same degree as the moral and self-regulatory domains (Gurven et al., 2013), intellectual efforts may be associated with attractiveness in many cultures. The increased likelihood of survival enjoyed by individuals with highly exploratory tendencies might be rooted in recognition of their overall creativity, which could implicate inquisitive individuals as possessing greater capacity for solving problems, including those related to effective parental investment (McCrae, 1987). Creativity seems to be deemed attractive (Haselton and Miller, 2006; Kaufman et al., 2008), and there is converging evidence that men and women focused on long-term mating motivations become particularly creative (Griskevicius et al., 2006) and are desirable in that context (Brown et al., 2020).

## Reciprocity of the Virtues

In discussing the evolutionary importance of the three virtue domains, McGrath (in press) discussed a concept first proposed by Aristotle usually referred to as the reciprocity of the virtues, suggesting a person would need to demonstrate a commitment to the entire array of virtues to be considered a relatively virtuous person. It is noteworthy that while the idea is attributed to Aristotle, he did not demonstrate reciprocity among the entire set of virtues he listed. For example, is it really the case that a person could not be deemed high in virtuousness if they are not munificent, even if munificence is a highly valued attribute?

McGrath suggested that virtues founded on abilities that have significant evolutionary value are likely to prove central to the judgment of someone as a globally virtuous individual. “The person who is productive but callous, the kind-hearted person who cannot be trusted to follow through, the accomplished person who refuses to challenge their beliefs no matter what evidence–none of these individuals meet the ideal of good citizenship, good fellowship, or living the right way, because they ultimately fail as a paragon for what is most helpful for the flourishing of the community” (McGrath, in press, p. 9).

This discussion suggests an empirical test for whether a certain virtue should be strongly considered in judgments about a high degree of virtue in an individual, i.e., which virtues should be considered reciprocal in judgments of self or others. If a virtue requires attributes identifiable in a wide variety of species, especially if there is evidence of convergent evolution (independent evolution in different species) of those attributes, that evidence supports the conclusion that the virtue should be given serious consideration as one needing to be present in an individual to a marked degree before that person could be considered high in virtuousness. Similarly, virtues considered in many cultures to be necessary for identifying someone as high in virtuousness are likely to demonstrate evolutionary precursors in other species. The determination of which virtues should be considered reciprocal has at least one valuable application, which is the identification of a set of virtues that should be encouraged in any program of character or virtue education.

## Practical Wisdom

One of the defining characteristics of an Aristotelian virtue ethics is the prominence allocated to the concept of practical wisdom or *phronesis*. Practical wisdom has to do with the capacity to deliberate effectively on the appropriate application of the virtues in specific contexts, including balancing the virtues, i.e., the pursuit of virtue in effective ways across situations and settings. Although enumerated among the Aristotelian virtues, practical wisdom is also seen as the organizing principle for all virtues through which the pursuit of goodness can be maximally effective. It is one of Aristotle’s intellectual virtues but helps mold how the highly virtuous person pursues the moral virtues.

Even without the Aristotelian context, it seems reasonable to hypothesize that the ability to apply principles of socially desirable behavior in ways that are optimal to the situation would be an indication of wisdom^4^. The central value of practical wisdom might suggest it as a, perhaps the, cardinal trait. This could be taken as implying a parallel between practical wisdom and the general factor in intelligence or personality (Littlefield et al., in press). We believe such a model is potentially defensible, but it would represent a variation from normal taxonomic practice, where hierarchies are based on overlapping features among subordinate elements. The relationship between practical wisdom and other virtues might better be understood in the relationship between mathematics and scientific disciplines. Mathematics shapes the activities in those other disciplines in very important ways, but it is not hierarchically superordinate to them in the way that concepts such as “social sciences” or “life sciences” would be.

The VIA Classification does not include a conceptualization of practical wisdom, but McGrath (2018) recently suggested it can be understood as the compound operation of three VIA character strengths: prudence, perspective, and judgment. Prudence has to do with the ability to delay acting impulsively in order to reflect more deeply on the situation and one’s emotional reactions to the situation. In fact, the term *phronesis* has sometimes been translated as prudence rather than as practical wisdom (e.g., Bartlett and Collins, 2011).

However, prudence by itself seems to be an incomplete representation of what is involved in practical wisdom. The individual needs to use both judgment and perspective in choosing the best course. The former has to do with identifying critical details of the situation necessary for making the best choice, the latter with the ability to see the situation in a larger context of more global considerations, including the moral background to the situation. This model would suggest practical wisdom requires delaying a response until deliberation on the best response has occurred (a self-regulatory skill), and deliberating on both situational and global factors as determinants of that best response (intellectual activities). We are therefore proposing practical wisdom as a composite of abilities bridging the self-regulatory and intellectual domains.

No empirical evidence currently exists to support this decomposition of practical wisdom. However, this formulation is markedly similar to a conceptualization of *phronesis* developed independently at the Jubilee Center for Character and Virtues (Darnell et al., 2019). Table 2 provides a comparison of the two models. While the concepts of prudence and emotion regulation are not equivalent, both have to do with emotional self-control appropriate to the situation. There is substantial overlap between the VIA judgment strength and the constitutive function in the Jubilee model, and between perspective and the integrative function. Finally, both models include the consideration of moral issues, though the model based on VIA strengths treats that as an aspect of perspective.

**TABLE 2:** Comparison of two formulations of practical wisdom.

**McGrath (2018)**	**Darnell et al. (2019)**
Prudence: “You are wisely cautious; you are planful and conscientious; you are careful to not take undue risks or do things you might later regret”	Emotion regulation: “*Phronesis* requires, and contributes to, the agent’s emotions being in line with her construal of a given situation, moral judgment, and decision” (pp. 119–120)
Judgment: “You examine things from all sides; you do not jump to conclusions, but instead attempt to weigh all the evidence when making decisions”	Constitutive function: “enables an agent to perceive what the salient features of a given situation are from an ethical perspective, and to see what is required in a given situation as reason(s) for responding in certain ways” (p. 118)
Perspective: “You take the ‘big picture’ view of things”	Integrative function: “involves integrating different components of a good life, especially in dilemmatic situations where different ethically salient considerations, or different sorts of virtue, appear to be in conflict” (p. 118)
	Moral blueprint: “Phronetic persons possess a general conception of living well (eudaimonia) and adjust their moral identity to that blueprint” (p. 119)

*Quotes describing the VIA strengths in the left column come from a questionnaire called the Global Assessment of Character Strengths (McGrath, 2019, p. 51), quotes in the right column from Darnell et al. (2019).*

One final point is worth making about potential contributions to a science of practical wisdom, which is that the concept clearly overlaps with other more traditional foci of psychological research such as judgment and problem-solving, and it may be valuable to mine these literatures to enhance the understanding of *phronesis*. For example, decision-making competence (Fischhoff, 2010) and complex problem-solving skills (Stadler et al., 2015) have both been found to correlate about 0.50 with measures of cognitive ability, a substantial relationship. At the same time, Fischhoff reported competence was also associated with higher socioeconomic status, absence of paternal substance use, and a more positive peer environment even after controlling for cognitive variables, suggesting better environmental circumstances can contribute to better decision-making skills (also see Odom, 1967). This finding suggests potential value in looking at relationships between practical wisdom and adverse childhood experiences (Felitti et al., 1998). There are some exciting possibilities here for integrating ancient insights with cutting edge topics.

## Conclusion

This article provides an initial effort to explore some of the ways in which the VIA Classification can be used to advance empirical investigations into the psychology of virtue. As noted previously, this is not intended to imply that the VIA Classification is a final system for understanding the character strength space. However, given the relative care associated with its development, it provides at least a very useful practical tool for testing hypotheses about this important concept.

We reviewed several lines of research and theorizing that can potentially contribute to progress in a science of virtue. First, no evidence exists to date suggesting that virtue is a state achieved. This finding, if replicated, may be taken as evidence that a life of virtue requires a continuing commitment to resisting temptation, thinking clearly when making one’s decisions, and even continued growth as a person who tries to do well by others while living well. Second, the elements of a relatively virtuous life tend to cluster into at least three categories, reflecting moral, self-regulatory, and intellectual functioning. This is not intended to represent a complete taxonomy, but in any attempt to draw comprehensive conclusions about virtue it probably would be best to evaluate whether those conclusions apply at least to these three constellations of virtues. Third, substantial evolutionary evidence is available suggesting the human capacity to act in ways concordant with these virtues is the product of multiple adaptations, each of which have contributed to the viability of species, with special attention paid here to reproductive viability. This feature of the virtues suggests that judgments about our virtue and the virtuousness of others should consider all three domains, rather than focusing exclusively on issues such as productivity or moral intent. Finally, we offer a model of practical wisdom as the combined use of three character strengths (prudence, judgment, and perspective) in a manner that potentially maximizes our effectiveness in problem-solving and decision-making. There is evidence to suggest that the capacity for practical wisdom correlates substantially with intelligence, but also with stability in personal background. This last finding supports the potential for uncovering other environmental determinants of practical wisdom.

Interest in a science of virtue is just emerging, and we stand at a starting point. We look forward to further tests of the hypotheses we have presented in this article, and hope it will inspire others to pursue those tests. In particular, as noted previously, initial efforts in this direction owe a strong debt to Western philosophy generally, and Aristotelian thinking more specifically. In attempting to expand upon the science of virtue more broadly, greater consideration should be given to non-Western perspectives on concepts consistent with the topic of virtue. That said, it is possible that other conceptions will so markedly differ from Western perspectives focusing on person-in-society that they should be considered distinct topics for study.

## Author’s Note

RM is a Senior Scientist for the VIA Institute, the copyright holder for the instruments discussed in this manuscript. The research summarized in this article was funded in part by the VIA Institute on Character. This work is part of a larger project done in collaboration with the Center for Character and Citizenship at the University of Missouri, St. Louis, and was made possible through the support of a grant from the John Templeton Foundation. The opinions expressed in this publication are those of the authors and do not necessarily reflect the views of the John Templeton Foundation or VIA Institute on Character.

## Author Contributions

RM provided oversight and primary authorship of most sections. MB provided primary authorship for the section on evolution and reviewed all sections. Both authors contributed to the article and approved the submitted version.

## Conflict of Interest

RM is a Senior Scientist for the VIA Institute on Character, which is the copyright holder for the VIA Classification. The remaining author declares that the research was conducted in the absence of any commercial or financial relationships that could be construed as a potential conflict of interest.

## Footnotes

1 See also https://www.virtuesproject.com/virtuesdef.html.

2 Jayawickreme and Fleeson (2017) and Miller (2017) have similarly raised questions about the existence of a distinctly virtuous class of individuals from a more conceptual perspective.

3 That said, the term *fundamental* was used to avoid implications that these three domains will necessarily be valued in all social orders, i.e., to avoid an unverifiable claim of essentialism as virtue concepts (Snow, 2019).

4 We will note this perspective on wisdom reflects the Aristotelian assumption that the height of wisdom has to do with effective engagement in one’s community. For example, the Buddhist conception of *prajñâ* is often translated as wisdom, but mainly has to do with achieving a deeper truth about reality that encourages detachment from material pursuits.
